# Meta-Analysis of Ocy-454 Showed Interrupted Osteocyte Maturation in Spaceflight Affects SOST Expression and Hypoxic Response

**DOI:** 10.3390/jcm14228100

**Published:** 2025-11-15

**Authors:** Mayuka Honjo, Takanori Hasegawa, Masafumi Muratani, Hiroki Bochimoto

**Affiliations:** 1 Division of Aerospace Medicine, Department of Cell Physiology, Jikei University School of Medicine, Tokyo 105-8461, Japan; 210077ms@tmd.ac.jp; 2 Department of Integrated Analytics, M&D Data Science Center, Institute of Integrated Research, Institute of Science Tokyo, Tokyo 113-8510, Japan; hasegawa.t.d968@m.isct.ac.jp; 3 Department of Genome Biology, Institute of Medicine, University of Tsukuba, Tsukuba 305-8572, Japan; muratani@md.tsukuba.ac.jp

**Keywords:** osteocyte, spaceflight, SOST, hypoxia, Sp7

## Abstract

**Background/Objectives**: Changes in sclerostin expression regulated by SOST in osteocytes during spaceflight may be associated with bone loss; however, the underlying mechanisms remain unclear. The aim of this study was to clarify the relationship between SOST expression and bone loss by identifying the gene expression differences between osteocytes with high and low SOST expressions. **Methods**: We used the NASA GeneLab Database OSD-324, which is a microarray of data about the Ocy454 osteocytic cell line cultured for 2, 4, and 6 days during spaceflight, and the GSE102958 microarray in the Gene Expression Omnibus. We also analyzed the characteristics of SOST gene expression in osteocytes during spaceflight using merged datasets. **Results**: The findings of Gene Set Enrichment Analysis (GSEA) revealed that five gene sets related with H3K27me3 significantly upregulated with NES > 2.0 during spaceflight compared with ground controls. We also found 77 and 617 differentially expressed genes (DEGs) in flight 6d vs. low and high SOST expression, respectively. We used the transcriptional regulatory relationships unraveled by the sentence-based text-mining (TRRUST) database to determine the most significant upstream transcription factor (TF) of genes downregulated in osteocytes during spaceflight compared with those expressing abundant SOST. We detected that TF Sp7 is the most significant, with FDR < 0.01. Moreover, the GSEA findings indicated that the hypoxic pathway is prolonged in osteocytes during spaceflight compared to those at ground level. **Conclusions**: The gene expression profiles of osteocytes during spaceflight and in comparatively immature osteocytes with low SOST expression were similar. Furthermore, early osteocyte maturation inhibited by downregulated Sp7 during spaceflight extended the hypoxic response.

## 1. Introduction

The space environment significantly differs from that of the Earth due to microgravity, radiation, and other factors. Since all life on Earth has adapted to gravity, microgravity induces a great impact on biological processes. For example, microgravity shifts body fluids upwards, which leads to compensatory changes in the cardiovascular system and causes atrophy of the musculoskeletal system that maintains a dynamic remodeling response to mechanical stimuli [1].

The effects of spaceflight on skeletal health present a significant challenge to long-term spaceflight. Bone loss in weight-bearing regions during spaceflight exceeds that among post-menopausal osteoporotic women, and the impact continues even after repatriation [2]. One cause of this is decreased bone formation by osteoblasts and increased resorption by osteoclasts determined by serum markers [3].

Bone consists of osteoclasts, osteoblasts, and osteocytes. Osteocytes account for 90% of bone cells and consist of fully differentiated osteoblasts that reside in the matrix of mineralized bone. Osteocytes control the responses of osteoclasts and osteoblasts to hormone signaling and mechanical stimuli via unknown mechanisms. However, osteocyte dendrites are considered to sense fluid flow inside the lacuna–canalicular system around osteocytes [4]. Osteocytes coordinate the activity of osteoclasts that comprise a major source of the receptor activator of osteoprotegrin (OPG) and the nuclear factor kappa beta ligand (RANKL) for osteoclastogenesis. Osteoprotegrin is a soluble decoy receptor allowing RANKL to inhibit osteoclast formation. Osteocytes contribute to osteoclast differentiation by secreting macrophage colony-stimulating factor (M-CSF), interleukin 6 (IL-6), and tumor necrosis factor-alpha (TNFα). Furthermore, osteocytes are a major source of sclerostin (encoded by the SOST gene), which plays a major role in bone homeostasis, as a negative regulator of the Wnt/β-catenin signaling pathway, and Dickkopf WNT signaling pathway inhibitor 1 (Dkk1) [5].

Osteocytes might release SOST in response to mechanical stimuli and regulate bone homeostasis. The expression of SOST increases in osteocytes during conditions of unloading and simulated microgravity [6,7], decreases under fluid shear stress [8] and loading [6], and mitigates bone loss induced under unloading caused by anti-sclerostin antibodies [9]. These findings indicate that osteocytes express SOST. Furthermore, osteocytes expressing high, rather than low, SOST [10] in response to mechanical stimulation are more differentiated.

Imbalanced bone remodeling during spaceflight might be associated with changes in osteocytes, because they control osteoclast activities. However, changes in SOST expression and sclerostin levels in spaceflight await clarification. On the one hand, increased SOST expression in osteocytes from goldfish scales in space is consistent with the findings in simulated microgravity at ground level [11]. On the other hand, although cranial bone density decreases [12] or does not significantly change during spaceflight [13], SOST increases [13], but serum sclerostin values do not significantly differ after 4-5 months of spaceflight [14]. The relationship between bone loss and SOST expression does not necessarily match findings on the ground from this perspective. In addition, whether SOST expression is altered in long bone osteocytes remains unknown. Thus, changes in SOST/sclerostin in spaceflight require further investigation.

We aimed to clarify whether SOST expression changes in osteocytes during spaceflight. We used OSD-324, which comprises the only experimental microarray information about osteocytes during spaceflight. The data were based on the osteocytic cell line Ocy454 derived from long bone that expressed SOST. Previous PCR findings found that SOST was downregulated in Ocy454 [15]. We combined the OSD-324 data with the GSE102958 microarray dataset that includes information about differences between osteocytes with low and high SOST expression [10]. We then analyzed characteristics of SOST gene expression in Ocy454 during spaceflight.

## 2. Materials and Methods

### 2.1. Data Download and Preprocessing

We downloaded DNA microarray datasets from the NASA GeneLab Data Repository (https://genelab-data.ndc.nasa.gov/genelab/projects/) (accessed on 17 September 2025) [16,17] and the National Center for Biotechnology Information (NCBI) Gene Expression Omnibus (GEO) database [18]. Specifically, we downloaded tape archive (tar) files in OSD-324 [15] (NASA GeneLab) and in GSE102958 [10] (NCBI GEO database) containing data about the murine osteocytic cell line Ocy454 [7]. The OSD-324 dataset included six spaceflight and ground control samples, each based on the GPL20258-[MTA-1_0] Affymetrix Mouse Transcriptome Array 1.0 [19]. We selected two samples each from the six spaceflight and ground (control) samples that were cultured for 2, 4, and 6 days. Two and three samples of Ocy454 expressing high and low SOST/sclerostin after 7 days of culturation were, respectively, downloaded from the GSE102958 dataset based on the GPL17791 [MoGene-2_0-st] Affymetrix Mouse Gene 2.0 ST Array [20].

The probe IDs of the dataset were converted into ENTREZID using mta10transctiptcluster.db [21] for OSD-324 and mogene20sttranscriptcluster.db [22] for GSE102958. We then filtered out NA data and ENTREZID duplicates using an EXCEL function [23] and merged these data using the dplyr version 1.1.3 package in R 4.4.1 [24]. We used RStudio version 2024.09.0.375 [25] for the R consoler.

### 2.2. Statistical Analysis (Differential Expression Analysis)

All data were statistically analyzed using Local Pooled Error (LPE) in R [26] and p-values were adjusted using the BH procedure, and FDR was calculated with the LPE package [27]. We considered any gene with FDR < 0.01 a differential expression gene.

### 2.3. Data Visualization

Data were visualized using principal component analysis (PCA), the prcomp function in R [26], and the Matplotlib [28] and Pandas modules [29] in Python 8.27.0 [30].

Chemical and genetic perturbations (CGP) of the curated gene sets in the Molecular Signatures Database (MSigDB) [31,32,33] were identified using Gene Set Enrichment Analysis (GSEA). Enrichment determined using GSEA 4.3.3 software [31,34] was considered significant if the absolute value of the normalized enrichment score (NES) was >2.0. Responses to hypoxia determined by GSEA were extracted using the CGP dataset and visualized using the clusterProfiler 4.12.6 package [35,36] in R. We also analyzed Kyoto Encyclopedia of Genes and Genomes (KEGG) [37,38,39] pathway enrichment using clusterProfiler and visualized the findings using the Matplotlib 3.9.2 and Pandas 2.2.2 modules in Python [30].

Volcano plots with fold change (FC) and false discovery rate (FDR) cut-offs of 0.5 and 0.01, respectively, were generated using the Matplotlib and Pandas modules in Python. We also extracted common differentially expressed genes (DEGs) using Venn diagrams generated using the matplotlib module [28] in Python.

We investigated DEGs using Gene Ontology (GO), the clusterProfiler package in R, and the GO.db 3.19.1 [40] R/Bioconductor package. Values with *p* < 0.05 were considered statistically significant, and were adjusted using the Benjamini–Hochberg (BH) procedure. We included the Biological Process (BP), Cellular Component (CC), and Molecular Function (MF) categories in GO.db, and visualized the data using Matplotlib in the Python Pandas module.

We identified upstream transcription factors among the DEGs using transcriptional regulatory relationships unraveled by sentence-based text-mining (TRRUST) [41]. Values with *p* < 0.01 were considered statistically significant.

## 3. Results

### 3.1. Gene Expression Profiles in Osteocytes Were Comparable After Six Days of Spaceflight and Low SOST Expression

**Figure 1:**
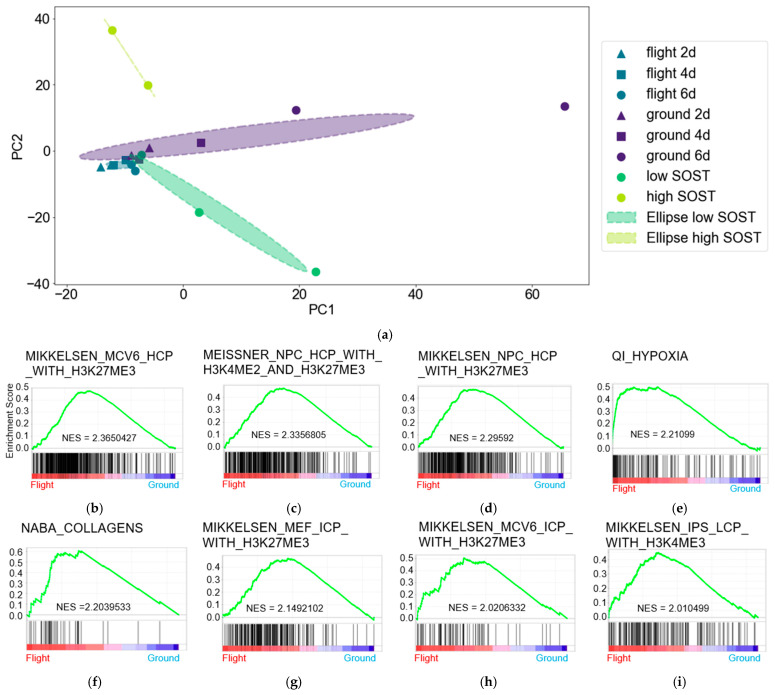

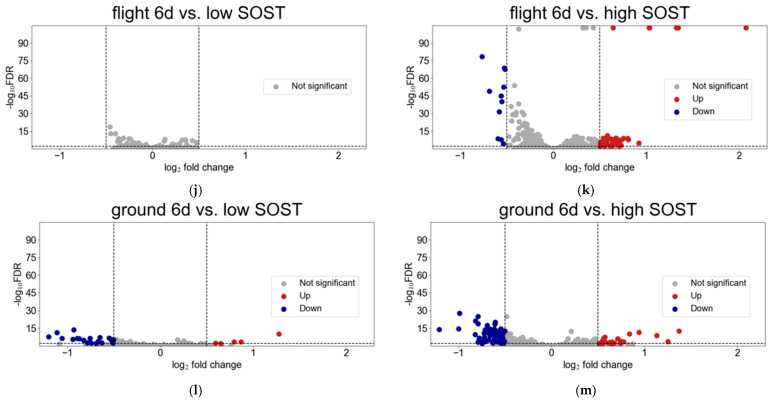
(**a**) General characteristics of samples determined using PCA. PC1 and 2 are principal components 1 and 2, respectively. (**b**–**i**) Significant GSEA findings using CGP dataset of flight vs. ground (NES > 2.0). (**j**–**m**). Volcano plots of (**j**) flight 6d vs. low SOST, (**k**) flight 6d vs. high SOST, (**l**) ground 6d vs. low SOST, and (**m**) ground 6d with high SOST. Fold change cutoff: 0.5. FDR cutoff: 0.01 for all. Flight 2d, 4d, and 6d: spaceflight for 2, 4, and 6 days. Ground 2d, 4d, and 6d: ground level for 2, 4, and 6 days.

We evaluated the characteristics of samples in the merged dataset using PCA [Figure 1a]. First, the samples obtained from OSD-324 include those cultured for 2, 4, and 6 days in both spaceflight and ground conditions. In addition, the samples obtained from GSE102958 include groups with low and high SOST expression levels after 7 days of culture. Spaceflight (flight) and ground samples as well as those with low and high SOST expression each formed independent clusters. Closer clusters were formed between time points by flight, than ground samples. The ground samples at 2 and 4 days, and the flight samples at 2, 4, and 6 days, each formed independent but close clusters. In contrast, ground samples formed separate clusters with each other or with flight samples at 6 days. Therefore, gene expression profiles differed between flight and ground samples at 6 days.

We investigated perturbed spaceflight-specific gene expression between flight vs. ground using GSEA and the CGP dataset, which provides information about chemical and genetic perturbation [Figure 1b–i]. Among eight significantly enriched genes, five were associated with H3K27ME3 histone modification in the flight and were compared with ground samples. Enhanced histone modification by H3K27ME3 indicated poorly differentiated cells [42] during spaceflight.

We compared the numbers of DEGs and changes in volcano plots to assess the similarity of SOST gene expression between flight 6d and poorly differentiated osteocytes. We detected 77 and 617 DEGs in flight 6d vs. low and high SOST expressions [Tables S1 and S2]. The volcano plots revealed significant differences in gene expression; they revealed that the most significantly different genes were concentrated at the center of flight 6d vs. high SOST expression and others scattered from the center [Figure 1j,k]. In contrast, the volcano plot showed greater fold changes among the genes in ground_6d vs. low, than in the high SOST expression [Figure 1l,m]. This suggested SOST expression was similarly low between flight 6d and ground osteocytes, and intermediate between osteocytes at ground_6d and those with low and high SOST expression.

### 3.2. Ossification Is Downregulated in Osteocytes After Spaceflight in Contrast to Those with High SOST Expression

**Figure 2:**
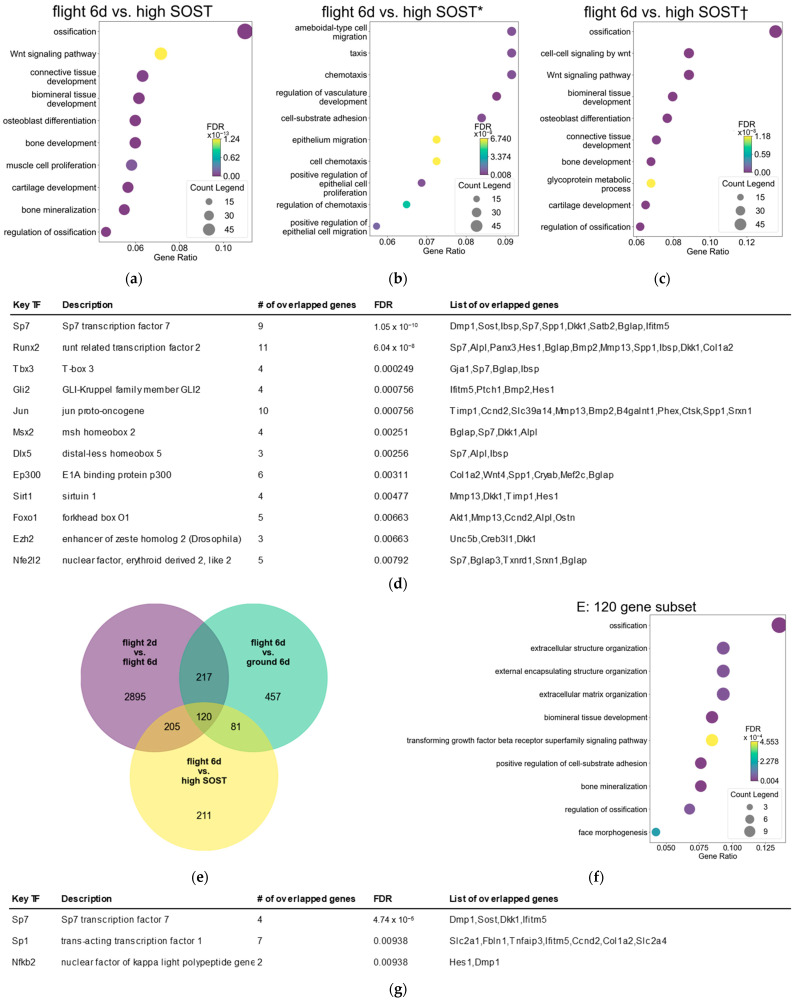
Gene Ontology analyses of DEGs between flight 6d and high SOST (**a**) vs. total, (**b**) * upregulated, and (**c**) † downregulated DEGs. (**d**) Transcription factor upstream of DEGs is downregulated in Flight 6d compared to high SOST detected by TRRUST (FDR < 0.01). (**e**) Venn diagram of total DEGs in flight 6d vs. 2d, flight 6d vs. ground 6d, and flight 6d vs. high SOST. (**f**) Gene Ontology analysis of DEGs extracted by Venn diagram (**e**). (**g**) Transcription factor upstream of genes extracted by Venn diagram in Figure 2e detected by TRRUST (FDR < 0.01). TF: transcription factor. #: number.

Gene expression was similar between flight 6d osteocytes and those with low SOST expression. Therefore, we compared osteocytes at flight 6d to those with low and high SOST expression, using GO [Figure 2a–c]. The GO map of DEGs in flight 6d vs. high SOST expression revealed enriched “ossification”, “Wnt signaling”, “biomineral tissue development”, and “osteoblast differentiation”, and “bone development” pathways [Figure 2a].

A GO map of DEGs downregulated in flight 6d compared to high SOST expression overlapped with “ossification”, “Wnt signaling pathway”, “cell–cell signaling by Wnt”, “biomineral tissue development”, “osteoblast differentiation”, and “bone development” pathways [Figure 2b]. A GO map of DEGs in flight 6d compared to high SOST expression revealed enriched “chemotaxis”,” taxis”, “ameboidal-type cell migration”, “cell–substrate adhesion”, and “cell chemotaxis” pathways associated with cell adhesion [Figure 2c]. These results indicated a significantly downregulated pathway that enhances ossification in flight 6d, compared to high SOST expression.

We analyzed TFs upstream of downregulated DEGs in flight 6d compared to osteocytes with high SOST expression using TRRUST, and detected the bone-specific TFs, Sp7 and Runx2 [Figure 2d]. Furthermore, we extracted common DEGs in flight 2d vs. 6d, ground 6d, and high SOST expression, using a Venn diagram to identify changes in gene expression over time during spaceflight, and detected 120 genes [Figure 2e]. Gene Ontology analysis of these 120 genes revealed enriched ossification pathways in downregulated DEGs in flight 6d vs. high SOST expression [Figure 2f]. Moreover, the most significant upstream TF of these 120 genes detected by TRRUST was Sp7, whereas Runx2 was undetectable [Figure 2g]. These results suggested that disrupted Sp7 expression in spaceflight inhibited Ocy454 maturation, and downregulated SOST expression.

### 3.3. Hypoxic Pathway in Osteocytes Remained Enriched After Spaceflight Compared to Ground

**Figure 3:**
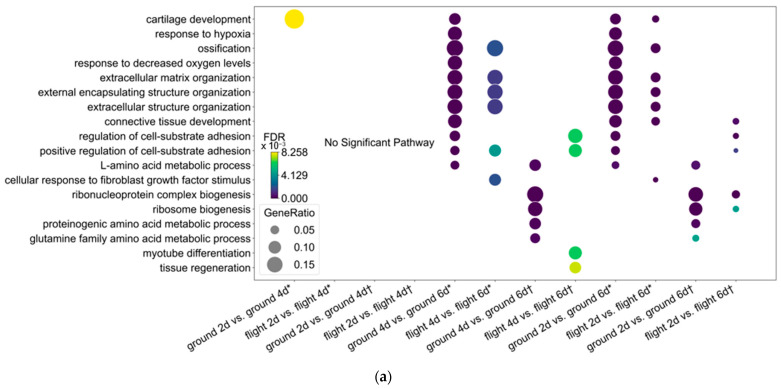

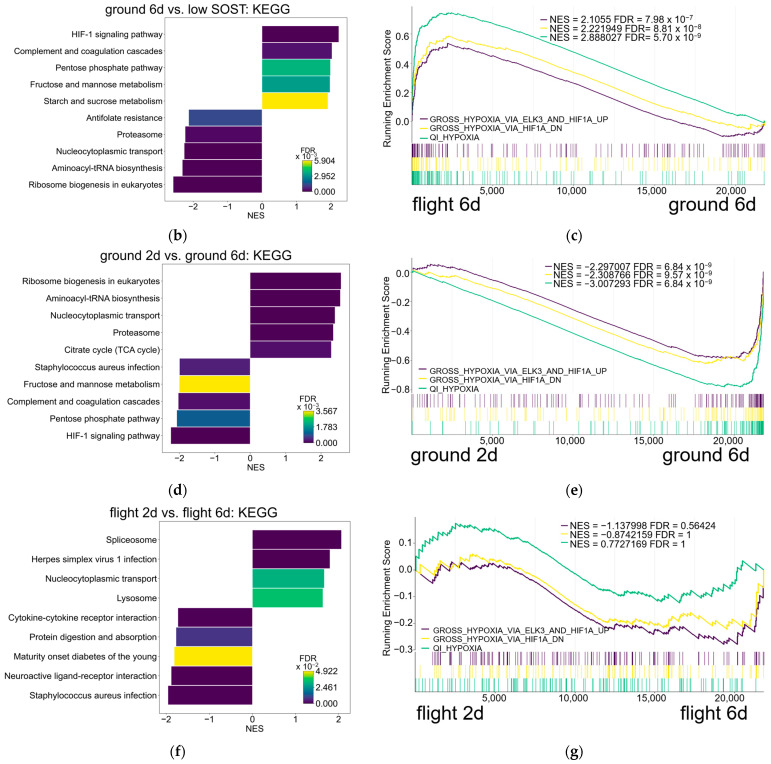
(**a**). Gene Ontology analysis of DEGs between timepoints vs. total, * upregulated, and † downregulated DEGs. Bar plot of top ten significant KEGG pathways of total DEGs in (**b**) flight 6d vs. ground 6d, (**d**) ground 6d vs. 6d, and (**f**) flight 6d vs. 2d. GSEA results of hypoxic pathways of total DEGs using CGP dataset for (**c**) flight 6d vs. ground 6d, (**e**) ground 2d vs. 6d, and (**g**) flight 6d vs. 2d.

We speculated that osteocyte maturation is inhibited in spaceflight based on the results of the comparison of osteocytes between flight 6d and those with high SOST expression. Therefore, we compared the temporal changes in flight versus on the ground using GO. The only significant pathway was “cartilage development” in downregulated DEGs in ground samples in flight d2 vs. d4 [Figure 3a]. These findings indicated that gene expression in osteocytes did not significantly change between 2 and 4 days regardless of being on the ground or in flight.

The pathways, “response to hypoxia” and “response to decreased oxygen levels” overlapped with downregulated DEGs in ground samples in 4d vs. 6d. However, these pathways were not enriched in flight [Figure 3a]. These similarly overlapped with downregulated DEGs in ground samples but not in flight 2d vs. 6d [Figure 3a]. This result indicated that the response to hypoxia was inhibited between 4 and 6 days in ground, but not in flight, samples.

We used KEGG enrichment to analyze the prolonged response to hypoxia in spaceflight determined by GO. The results of ground 6d vs. flight 6d revealed that “HIF-1 signaling”, the “pentose phosphate pathway”, and “fructose and mannose metabolism” associated with energy metabolism overlapped the upregulated DEGs in Flight 6d [Figure 3b]. Similarly, in ground 2d vs. 6d, the “HIF-1 signaling pathway” and pathways related to energy metabolism, such as “the pentose phosphate pathway”, “fructose and mannose metabolism”, and “protein digestion and absorption”, overlapped with downregulated DEGs in ground 6d [Figure 3d]. Contrary to these results found in flight 2d vs. 6d, pathways enriched in ground 6d vs. flight 6d and ground 2d vs. 6d were undetectable, whereas “herpes simplex virus 1 infection”, “cytokine–cytokine receptor interaction”, and “staphylococcus aureus infection” associated with infection were enriched [Figure 3f].

We applied GSEA to the CGP dataset in flight 6d vs. ground 6d [Figure S1] and found that “GROSS_HYPOXIA_VIA_ELK3_AND_HIF1A_UP”, “GROSS_HYPOXIA_VIA_HIF1A_DN”, and “QI_HYPOXIA” were upregulated in flight 6d with NES > 2.0 [Figure 3c]. These pathways were downregulated in ground 6d vs. 2d with NES < −2.0 [Figure 3e and Figure S2]. In contrast, the NES of these pathways was insignificant in flight 2d vs. 6d [Figure S3 and Figure 3g]. These results indicated that the hypoxic pathway is prolonged in osteocytes after spaceflight compared with those on the ground.

### 3.4. Osteocytes After Spaceflight Maintain Energy Metabolism Pathway Downstream of Hif1a and Egr1 Compared to Osteocytes with High SOST Expression

**Figure 4:**
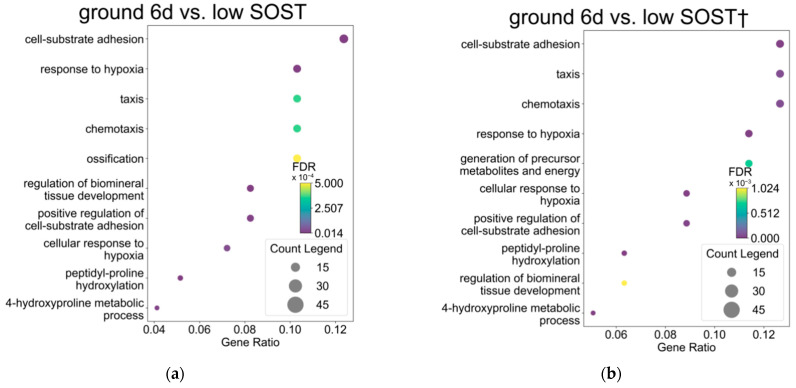

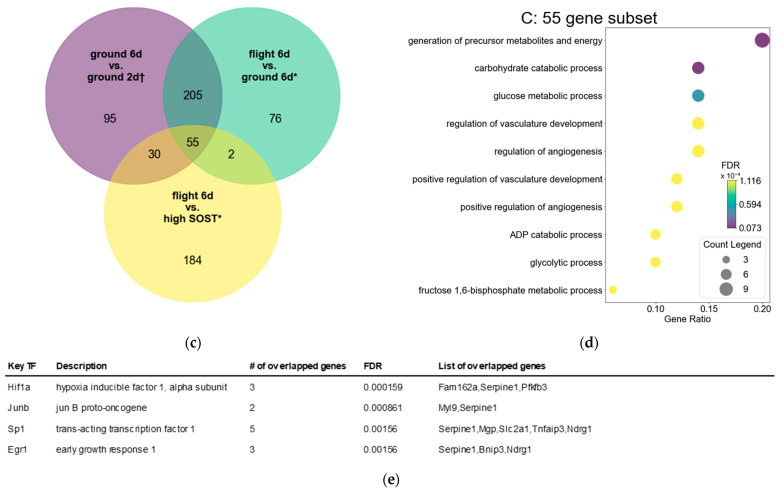
(**a**,**b**) GO analysis of total (**a**) and upregulated (**b**) DEGs in ground 6d vs. low SOST. (**c**) Venn diagram of DEGs upregulated in flight 6d vs. ground 6d (flight 6d vs. ground 6d *), and flight_6d vs. high SOST (flight_6d vs. high SOST †), and DEGs downregulated in ground 6d compared to ground 2d (showed as ground 6d vs. ground 2d †). (**d**) GO analysis of extracted 55 genes in Figure 4c Venn diagram (**e**). Transcription factor upstream of genes extracted by Venn diagram in Figure 4d detected by TRRUST (Key TF: Gene Symbol for Transcription factor, #: number) (FDR < 0.01).

We investigated whether the response to hypoxia is prolonged in osteocytes with low SOST expression as it is in flight 6d by analyzing DEGs using GO. All DEGs between ground 6d and low SOST overlapped with pathways associated with “response to hypoxia” and “response to oxygen levels” [Figure 4a]. The upregulated DEGs in low SOST expression vs. ground 6d overlapped with “response to hypoxia” and “cellular response to hypoxia” pathways [Figure 4b]. In contrast, significant pathways were not associated with downregulated DEGs in osteocytes with low SOST expression. These results indicated that the gene profile of “response to hypoxia” was prolonged in osteocytes on flight 6d and in those with low SOST expression.

We suggested above that the response to hypoxia is maintained in osteocytes during spaceflight, but downregulated in ground controls. We then considered whether the prolonged response to hypoxia and SOST expression is abnormal and specific to spaceflight. Therefore, we used Venn diagrams to extract common genes from DEGs that were upregulated in flight 6d vs. ground 6d and vs. high SOST expression, and downregulated in ground 2d vs. 6d. We found 55 responsive DEGs in flight 6d vs. high SOST expression [Figure 4c].

Gene Ontology analysis of the 55 responsive DEGs and pathways associated with energy metabolism revealed that “generation of precursor metabolites and energy”, “carbohydrate catabolic process”, and “glucose metabolic process” overlapped [Figure 4d]. Furthermore, TRRUST detected the upstream Hif1a and Egr1 transcription factors of these 55 genes involved in “responses to hypoxia” [Figure 4e]. These findings indicated that spaceflight conditions enhance energy metabolism via signaling pathways downstream of Hif1a and Egr1 that are associated with “response to hypoxia” in Ocy454 osteocytes.

## 4. Discussion

We found a similar gene expression between osteocytes after spaceflight and those with low SOST expression on the ground. The expression of a gene set induced by H3K27 trimethylation was increased, that of genes downstream of Sp7 was decreased, and pathways downstream of HIF-1a were increased compared to ground controls. The expression of HIF-1a decreased over time on the ground, but remained elevated during spaceflight. Consequently, the expression of genes associated with energy metabolism was enhanced. This is the first comprehensive meta-analysis to examine the effects of spaceflight on osteocyte gene expression with a focus on SOST.

The increase in the level of trimethylation of H3K27(H3K27me3) in osteocytes during spaceflight is considered to be related to a low osteocyte differentiation status according to former research. Expression of the H3K27me2/3 demethylase, ubiquitously transcribed tetratricopeptide repeat, X chromosome (Utx) is induced during osteocyte differentiation [43]. Excessive H3K27me3 prevents Msx-2 and Dlx-5 from approaching binding sites on the promoter regions of Runx2 and Sp7, which become downregulated [42]. This is consistent with the reduced expression of Sp7 downstream genes in osteocytes after spaceflight [Figure 2d]. Notably, SOST is downstream of Sp7, which is the only TF that controls endogenous sclerostin secretion.

The Sp7 TF is key to osteocyte differentiation and dendrite formation. The dendrite population is reduced in osteocytes that do not express Sp7 [44]. Although changes in dendrites during spaceflight have not been determined, osteocytes in suspension cultures have fewer dendrites [45]. Furthermore, Ocy454 cells acquired an early osteolytic expression profile within ~7 days of enculturation. The enculturation of OSD-324 and Ocy454 began five days before and two days after launch, respectively [15]. Therefore, the idea that spaceflight suppressed the formation of dendrites in early osteocytes is reasonable. Osteocytes in a hypoxic environment use dendrites to preserve metabolic capacity [46]. Thus, fewer dendrites induce an enhanced hypoxic response. This is consistent with our finding that the response to hypoxia remained elevated and enhanced energy metabolism. Taken together, whereas osteocytes on the ground continuously developed dendrites throughout culture, Sp7-mediated dendrite growth during spaceflight was inhibited. This prevented osteocytes from maintaining metabolism and resulted in a prolonged hypoxic response.

This study has several limitations. It should be noted that this study only examined overall trends in gene expression and did not include in vivo experiments using knockout mice or in vitro experiments using siRNA. The expression of SOST is also affected by induced random vibrations and lower CO_2_ levels. Such factors might impact SOST expression during spaceflight enculturation although it was decreased in the present study. We could not confirm whether H3K27me3 is enhanced in spaceflight because CHIP-seq was not available. We could not examine the morphology of osteocytes, which prevented us from confirming the numbers of dendrites in osteocytes during spaceflight. Morphological changes in osteocytes during spaceflight remain unknown and await further investigation. We combined data from OSD-324 osteocytes pre-cultured for 7 days followed by 2–6 days, and GSE102958 cultured for 7 days. Because these datasets included only two or three samples per condition, caution is warranted when generalizing our results. Nonetheless, we identified similar gene profiles between osteocytes in flight 6d and those with low SOST expression, downregulated genes downstream of Sp7, and a prolonged hypoxic response in osteocytes during spaceflight.

In this study, our findings suggest that the decrease in bone density under microgravity may be attributed not only to the regulation of osteoclast and osteoblast activity by secreted factors such as SOST, but also to the maturation process of osteocytes themselves. Furthermore, H3K27me3 and Sp7 may play important roles in this osteocyte maturation. Therefore, future studies should focus not only on osteocyte secretion but also on the maturation of osteocytes regulated by factors such as H3K27me3 and Sp7. This will be key to developing effective strategies for preventing bone loss during spaceflight and disuse syndrome on Earth.

From a clinical perspective, the molecular pathways identified in this study may also explain the bone fragility observed in patients with prolonged immobilization, paralysis, or age-related osteoporosis. Monitoring circulating biomarkers that reflect osteocyte maturation status—such as sclerostin or osteocyte-derived microRNAs—could provide a minimally invasive approach for assessing bone quality in these populations. Moreover, therapeutic interventions targeting the epigenetic regulation of osteocyte maturation (e.g., the modulation of H3K27me3) or combining such strategies with mechanical loading–based rehabilitation may help preserve bone strength both in space travelers and bedridden patients on Earth. Thus, insights from spaceflight research offer a promising translational framework for improving skeletal health in clinical settings.

## 5. Conclusions

In conclusion, we showed that spaceflight inhibits early osteocyte maturation by decreasing the expression of Sp7 downstream genes. This results in decreased SOST expression and an extended hypoxic response. These findings basically indicate that early osteocyte maturation is responsible for the decreased bone density caused by spaceflight. Furthermore, H3K27me3 and Sp7 play key roles in early osteocyte maturation and might serve as therapeutic targets for preventing bone loss during long-term human spaceflight. Changes in osteocyte morphology including dendrites during spaceflight require further investigation.

## Data Availability

All relevant data are within the manuscript and are fully available without restriction.

## Supplementary Materials

The following supporting information can be downloaded at: https://www.mdpi.com/article/10.3390/jcm14228100/s1, Table S1: DEGs in flight 6d vs. low SOST; Table S2: DEGs in flight 6d vs. high SOST; Figure S1: Significant GSEA result using CGP dataset of Flight 6d vs. Ground 6d (NES > 2.0); Figure S2: Significant GSEA result using CGP dataset of Ground 6d vs. Ground 2d (NES > 2.0); Figure S3: Significant GSEA result using CGP dataset of Flight 6d vs. Flight 2d (NES > 2.0).

## Abbreviations

The following abbreviations are used in this manuscript:

OPG	Osteoprotegrin
RANKL	Nuclear factor kappa beta ligand
M-CSF	Macrophage colony-stimulating factor
IL-6	Interleukin 6
TNFα	Tumor necrosis factor-alpha
Dkk1	Dickkopf WNT signaling pathway inhibitor 1
NCBI	National Center for Biotechnology Information
NASA	National Aeronautics and Space Administration
GEO	Gene Expression Omnibus
LPE	Local Pooled Error
BH	Benjamini–Hochberg
PCA	Principal component analysis
CGP	Chemical and genetic perturbations
MSigDB	Molecular Signatures Database
GSEA	Gene Set Enrichment Analysis
NES	Normalized enrichment score
KEGG	Kyoto Encyclopedia of Genes and Genomes
FC	Fold change
FDR	False discovery rate
DEGs	Differentially expressed genes
GO	Gene Ontology
BP	Biological Process
CC	Cellular Component
MF	Molecular Function
TRRUST	Transcriptional regulatory relationships unraveled by sentence-based text-mining
H3K27me3	The trimethylation of H3K27
Utx	X chromosome
