# Supplementary material for: Meta-Analysis of Ocy-454 Showed Interrupted Osteocyte Maturation in Spaceflight Affects SOST Expression and Hypoxic Response
Source: J Clin Med. 2025 Nov 15;14(22):8100. doi: 10.3390/jcm14228100 (PMC12653756; doi:10.3390/jcm14228100)
Supplement: Supplementary file 1 [file jcm-14-08100-s001.zip › supplementary_figure.pdf]

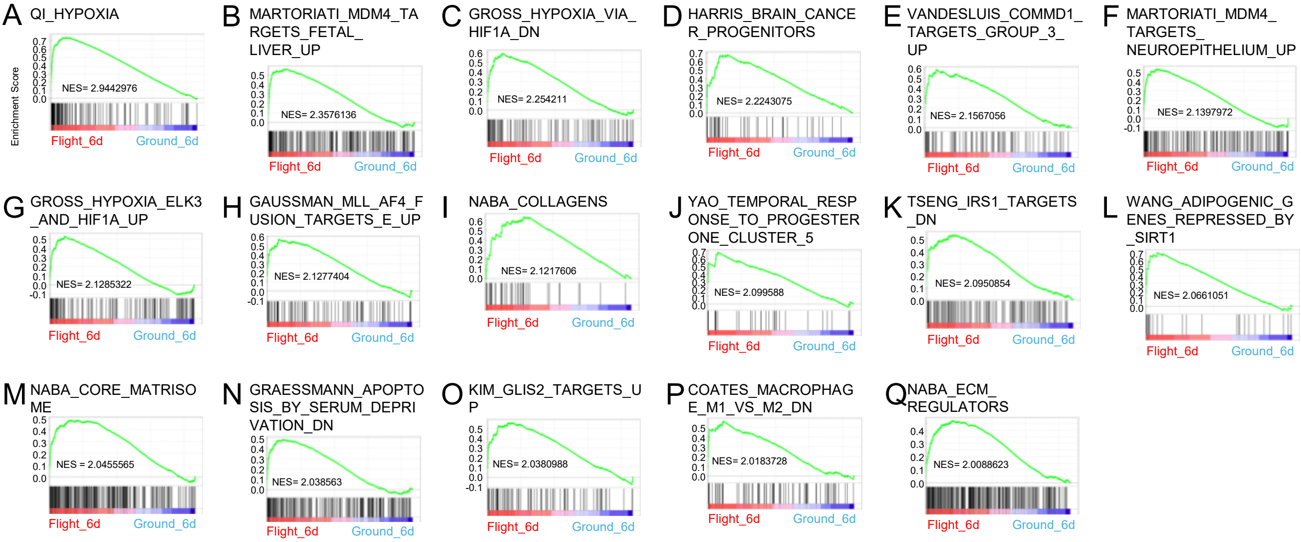

Supplementary Figure 1. Significant GSEA result using CGP dataset of Flight 6d vs Ground 6d (NES > 2.0).

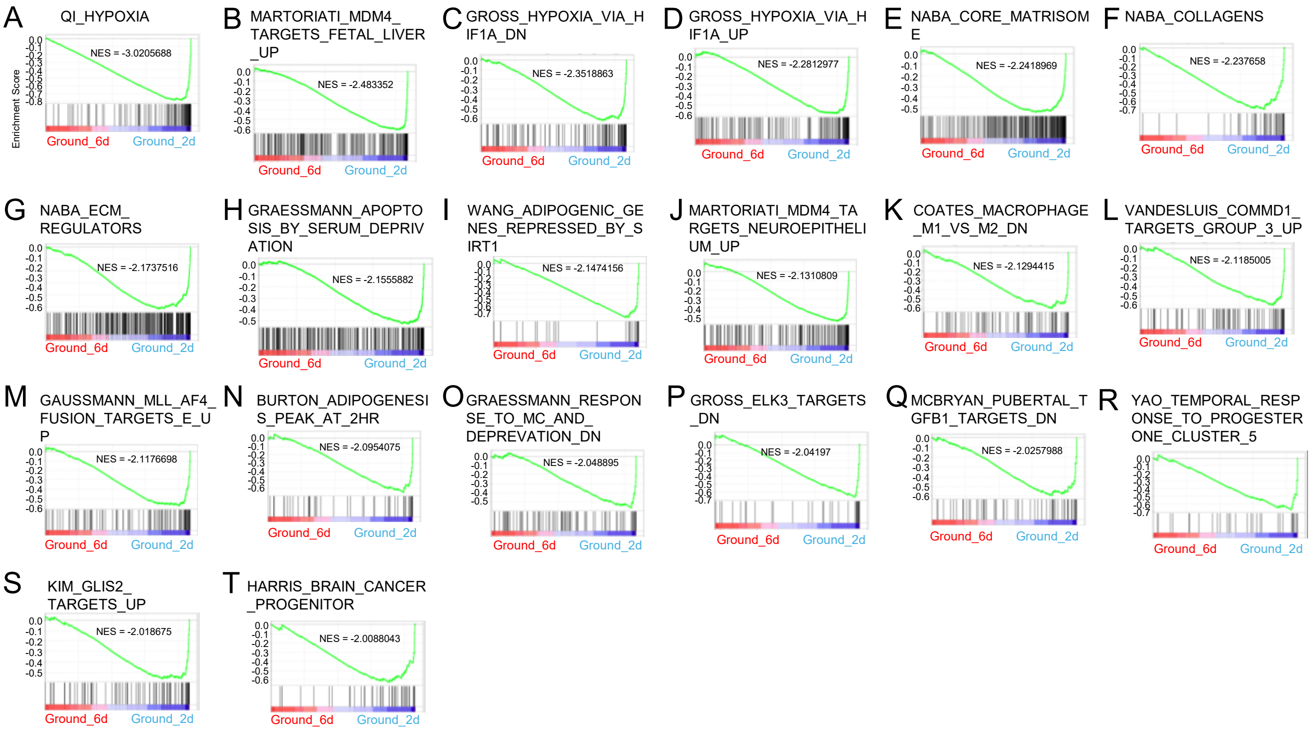

Supplementary Figure 2. Significant GSEA result using CGP dataset of Ground 6d vs Ground 2d (NES > 2.0).

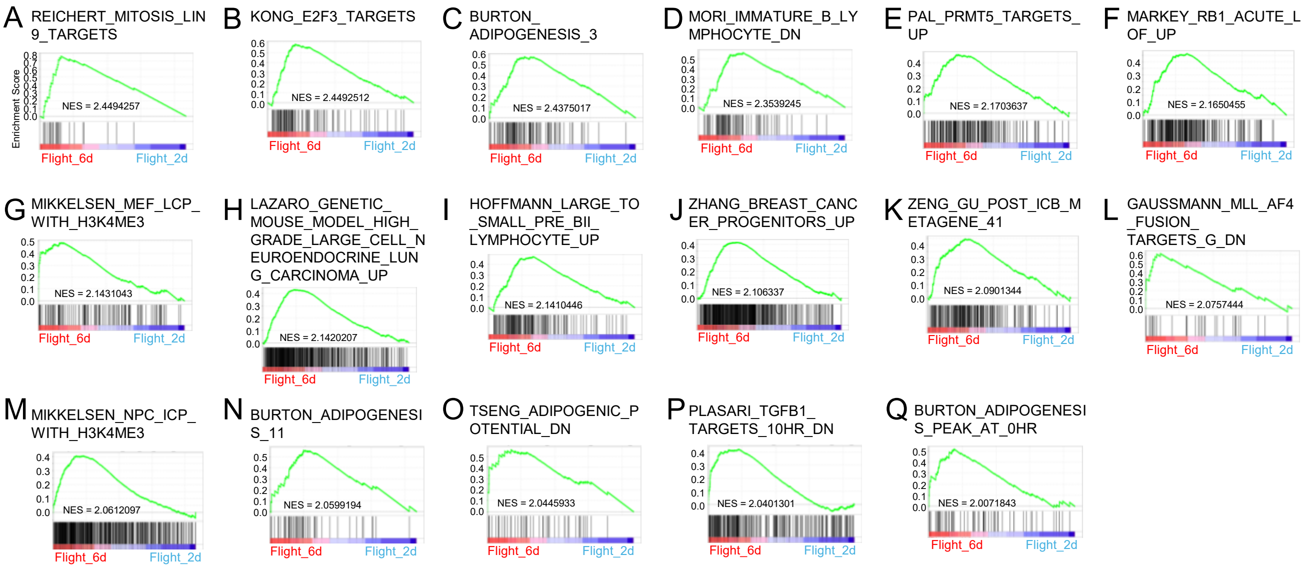

Supplementary Figure 3. Significant GSEA result using CGP dataset of Flight 6d vs Flight 2d (NES > 2.0).
